# Serum concentrations of different or multiple vitamins and Sarcopenia risk among US adults: insights from NHANES

**DOI:** 10.1186/s12889-024-20897-9

**Published:** 2024-12-04

**Authors:** Li Liu, Xueman Ding, Yue Zhang, Tingting Li, Panpan Xu, Yue Ma, Hengrui Xing, Qiang Niu, Mulatibieke Keerman

**Affiliations:** 1 Department of Preventive Medicine, School of Medicine, Shihezi University, North 2th Road, Shihezi, 832000 Xinjiang People’s Republic of China; 2 Key Laboratory for Prevention and Control of Emerging Infectious Diseases and Public Health Security, the Xinjiang Production and Construction Corps, Shihezi, Xinjiang People’s Republic of China; 3 Key Laboratory of Xinjiang Endemic and Ethnic Diseases (Ministry of Education), School of Medicine, Shihezi University, Shihezi, Xinjiang People’s Republic of China; 4 NHC Key Laboratory of Prevention and Treatment of Central Asia High Incidence Diseases, (First Affiliated Hospital, School of Medicine Shihezi University), Shihezi, Xinjiang People’s Republic of China

**Keywords:** Serum vitamins, Sarcopenia, Combined association, Nonlinear association

## Abstract

**Background:**

The relationship between serum concentrations of different or multiple vitamins and sarcopenia remains underexplored. This investigation evaluates potential links between serum concentrations of different or multiple vitamins and sarcopenia prevalence among adults in the United States.

**Methods:**

Utilizing a cross-sectional design, this research draws from the National Health and Nutrition Examination Survey (NHANES) dataset of 2003–2006, encompassing 5,060 participants with comprehensive serum vitamin A, E, B9, B12, C, and D concentrations, alongside sarcopenia and covariate measurements. Participant stratification into distinct vitamin co-exposure clusters was achieved through K-means clustering. Analytical models, including weighted logistic regression, restricted cubic splines (RCS), weighted quantile sum regression (WQS), quantile g-computation (Q-gcomp), and Bayesian kernel machine regression (BKMR), were employed to evaluate the association between serum concentrations of different or multiple vitamins and sarcopenia risk, with an emphasis on nonlinearity.

**Results:**

In this study, sarcopenia was detected in 681 individuals (13.46%). Logistic regression results did not demonstrate any linear association between individual vitamin levels and sarcopenia risk (P_FDR_ > 0.05). Contrastingly, the RCS model unveiled significant non-linear relationships for vitamins A and D (*P*_non-linear < 0.05). The K-means clustering results showed that participants in high-level vitamin exposure group had lower sarcopenia risk compared with those in low-level vitamin exposure group (OR (95% CI): 0.582 (0.397, 0.852)). Additionally, higher serum concentrations of different or multiple vitamins correlated inversely with sarcopenia risk (*P*_trend = 0.002). This inverse association was corroborated by WQS, Q-gcomp, and theBKMR models and remained consistent upon sensitivity analysis.

**Conclusions:**

This study elucidates an inverse correlation between serum concentrations of different or multiple vitamins and sarcopenia risk, emphasizing a non-linear association, particularly with suboptimal vitamin D concentrations. Given the limitations of the NHANES study, further researches are required to clarify the existence of these relationships.

**Supplementary Information:**

The online version contains supplementary material available at 10.1186/s12889-024-20897-9.

## Introduction

Sarcopenia, identified as a systemic and progressive skeletal muscle disorder, manifests through diminished muscle mass, reduced strength, and/or decreased physical performance [1]. Importantly, sarcopenia is closely associated with severe health consequences, including an elevated risk of falls, fractures, metabolic syndromes, and cognitive decline [2]. The global healthcare costs associated with sarcopenia may rise significantly due to its implications [3]. The prevalence of sarcopenia ranges from 10% in the general population to 10–27% among individuals aged 60 years or older in Europe [4]. Although some of the increased prevalence of sarcopenia can be attributed to population aging, sarcopenia has been identified in younger individuals recently [5, 6]. Given these implications, it is crucial to implement proactive measures aimed at preventing sarcopenia.

Vitamins are essential organic compounds that perform pivotal functions in metabolic pathways, cellular growth, and developmental processes [7]. Numerous studies have examined the impact of individual vitamins on sarcopenia, but the results have been inconsistent. For instance, animal-based research underscores vitamin D’s role in modulating muscle metabolism, which could potentially mitigate the decline in muscle function and mass associated with aging [8, 9]. Nonetheless, the data from several randomized controlled trials examining the efficacy of vitamin D supplementation in preventing the occurrence of sarcopenia have failed to establish a definitive link between vitamin D levels and the condition [10–12]. Similarly, studies examining the relationship between vitamins A, C, and E and the risk of sarcopenia have yielded inconsistent results [13–16]. This discrepancy may be attributed to reliance on self-reported nutritional data, which is susceptible to recall bias, rather than precise serum vitamin measurement [17]. Serum levels are a more objective measure of vitamin exposure when exploring associations with sarcopenia, but should also acknowledge here that they are influenced by other things beyond diet, such as supplement use and exposure to ultraviolet radiation.

Although research on the relationship between serum concentrations of different or multiple vitamins and sarcopenia is limited, the observed associations between individual vitamins and sarcopenia suggest that the potential interactions among these vitamins warrant further investigation. Furthermore, individuals in real life are often simultaneously exposed to multivitamins from diverse sources. Given the potential for non-linear correlations and possible interactions among vitamins, appropriate methods are essential when exploring the association between serum concentrations of different or multiple vitamins and the risk of sarcopenia. Weighted quantile sum regression (WQS), quantile-based g computation (Q-gcomp), and Bayesian kernel machine regression (BKMR) models are commonly utilized models for analyzing the relationship between mixed exposures and sarcopenia risk in recent years [18, 19], which can comprehensively assess the association between serum concentrations of different or multiple vitamins and the risk of sarcopenia, thereby enhancing the reliability of the research findings.

Our study utilized data from the National Health and Nutrition Examination Survey (NHANES) with the aim of elucidating the independent and synergistic associations of serum concentrations of different or multiple vitamins (A, E, B9, B12, C, and D) on sarcopenia risk among a diverse cohort of American adults aged 20 years and above. Prior research has indicated a protective association between multivitamin co-exposure and the risk of other diseases [20, 21]. Building on this foundation, we hypothesized that serum concentrations of different or multiple vitamins are negatively associated with the risk of sarcopenia. We employed a series of statistical methods to conduct a comprehensive analysis aimed at testing this hypothesis, with the hope of providing novel insights into prevention strategies for sarcopenia.

## Materials and methods

### Study population

The study utilized data from the NHANES, which is a program of studies designed to assess the health and nutritional status of adults and children in the United States. This survey, which is representative of the national population, receives approval and sponsorship from the National Center for Health Statistics (NCHS). Methodology involved participants undergoing comprehensive in-home interviews before they were invited for physical assessments at mobile examination centers (MECs). During these visits, certified medical personnel collected biological specimens, including blood and urine. The NHANES protocol was reviewed and sanctioned by the NCHS Ethics Review Board, ensuring adherence to ethical research practices. Informed consent was duly obtained in writing from all individuals prior to their participation.

**Fig. 1:**
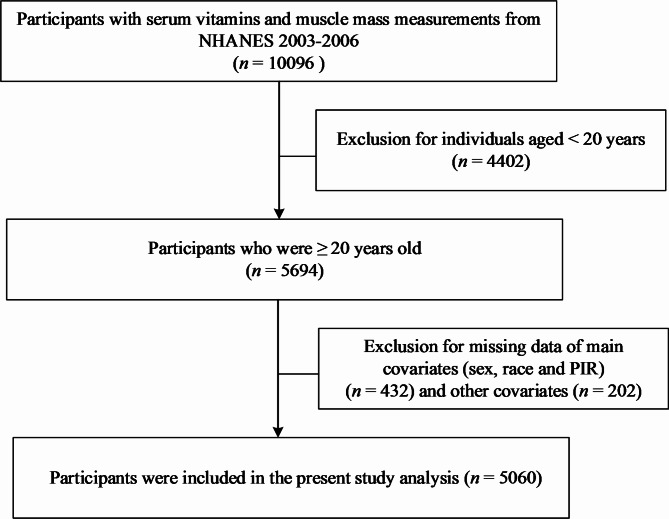
Flow diagram of the study population

The scope of this research was confined to data from the 2003–2006 NHANES cycles, a period during which both serum vitamin levels and muscle mass measurements were comprehensively recorded. Inclusion criteria mandated that subjects be U.S. residents aged 20 years or older. Our analysis was restricted to participants who had complete datasets for both the variables of interest - muscle mass and serum vitamin concentrations - and all covariates deemed relevant for this study to control for potential confounding factors. Following these stringent inclusion parameters, the final analytic sample comprised 5,060 adults. For a visual depiction of participant selection, please refer to the flow diagram presented in Fig. 1.

### Measurement of serum vitamins

Serum specimens, collected as part of the study protocol, were transported to the Division of Environmental Health Laboratory Sciences, which operates under the auspices of the National Center for Environmental Health at the Centers for Disease Control and Prevention (CDC) for detailed analyses. A total of six serum vitamins were assayed from samples obtained during the NHANES cycles spanning 2003 to 2006. The vitamins quantified included vitamin A (retinol), vitamin E (alpha-tocopherol), vitamin B9 (folate), vitamin B12 (cobalamin), vitamin C (ascorbic acid), and vitamin D (25-hydroxyvitamin D). Each vitamin’s assay followed standardized laboratory method protocols, with the detection limits (LOD) for these being readily obtainable from official sources. Consistent with NHANES guidelines, concentrations falling below the LOD were assigned a value calculated using the formula LOD divided by the square root of two. Table S1 elucidates the serum vitamin levels’ detailed distribution statistics.

### Sarcopenia

Sarcopenia was defined based on the guidelines established by the Foundation for the National Institutes of Health (FNIH) and was characterized by skeletal muscle mass adjusted by body mass index (ASM/BMI) < 0.512 for females and < 0.789 for males [22]. Dual-energy x-ray absorptiometry (DXA) was used to measure the muscle mass in the arms and legs, enabling the calculation of skeletal muscle mass. To ensure safety, individuals who were pregnant, with a body weight > 136 kg, or with a height > 196 cm were excluded from the DXA measurements.

### Covariates

Based on the literature, the following variables were considered as covariates. Demographic variables included age, sex, race (Mexican American, non-Hispanic black, non-Hispanic white, and Other), family income to poverty ratio (PIR), and education level (lower than high school, high school, or above high school). The BMI [weight (kg)/height (m^2^)] was calculated and classified as non-obese (< 30 kg/m^2^) or obese (≥ 30 kg/m^2^). Lifestyle variables included smoking status (never, former, or current) [23], drinking status (never, former, or current) [24], and physical activity level. Physical activity level was evaluated through the use of the Physical Activity Questionnaire and categorized into four groups (sedentary = no regular physical activity; Insufficient = < 500 MET-minutes per week; moderate = 500–1000 MET-minutes per week; and high = > 1000 MET-minutes per week) [25]. The presence of hypertension or diabetes was defined by participants’ self-reports of physician diagnoses. The serum total protein (g/dL) was measured using the Beckman Synchron LX20 instrument. This measurement includes both albumin (ALB) and globulin (GLB) and is closely associated with protein intake and immune function [26]. Energy intake (kcal) was obtained through dietary interviews with the subjects.

Moreover, we adjusted for the season of sample collection and the time of blood collection to account for the variations in serum vitamin levels throughout the day, week, and year [27]. The season of sample collection was coded corresponding to the examination date and was divided into two categories (November 1 to April 30, and May 1 to October 31). The time of venipuncture was classified into three sessions (morning, afternoon, and evening). Further details regarding the relevant covariates are available on the NHANES website.

### Statistical analysis

Demographic characteristics were represented as numbers (n) and percentages (%) for categorical variables, and mean and standard deviation (SD) for continuous variables. To compare the distribution differences of variables between the sarcopenia and non-sarcopenia groups, the t-test (normal distribution) or Mann-Whitney U test (skew distribution) was used for continuous variables, and the Chi-square test was used for categorical variables. The serum vitamin measurements were initially log-transformed to ensure a normal distribution and subsequently standardized to eliminate the impact of different units. Correlation between pairwise serum vitamin concentrations was assessed using Spearman correlation coefficients.

#### Multivariate logistic regression and RCS analysis

Weighted multivariable logistic regression was applied to explore the association between single vitamin levels and sarcopenia among participants. Vitamins were both fitted as log-transformed continuous variable and categorical variable to calculate odds ratios (OR)and corresponding 95% confidence intervals (CI). Tests for trend (*P* for trend) were conducted across quartiles using the median serum vitamins concentration in each quartile as a linear variable in the regression models. All *P*-values are further FDR corrected. Furthermore, we conducted the restricted cubic splines (RCS) analysis with 4 knots at the 5th, 35th, 65th, and 95th percentiles of log-transformed concentrations to explore the nonlinear relationship shape between each vitamin and sarcopenia. The reference point (OR = 1) was designated as the median value of serum vitamin levels. *P* for nonlinear was calculated through Wald testing for RCS coefficients.

#### K-means analysis

To investigate the relationship between six serum vitamin measurements and the risk of sarcopenia. First, K-means cluster method was employed to analyze and classify the serum vitamin concentration data, considering the mean silhouette width, elbow method, and biological interpretability [28]. The clustering results are shown in Fig S2. The association between the identified cluster patterns and sarcopenia risk was assessed by the prior multivariable logistic regression model.

#### Weighted quantile Sum regression

Then WQS regression was used to estimate the potential associations between multiple vitamin concentrations and sarcopenia as it combines linear or logistic regression with a mixed-effect strategy [29]. Notably, WQS regression constrained the association of individual vitamins on sarcopenia risk to remain in one direction. In this study, we established a WQS index based on quartiles of these vitamins. Bootstrap sampling (*n* = 1000) was used to determine the contribution weights of each vitamin to the overall impact of the mixture, with the weights summing up to 1. 40% of the data was used as the test set, while the remaining 60% served as the validation set. Given the limited research on the relationship between serum concentrations of different or multiple vitamins and sarcopenia, we employed the WQS to examine the bidirectional influence of the WQS index on the risk of sarcopenia.

#### Quantile g-computation

To investigate whether we observed consistent findings across different multi-vitamin approaches, we also performed Q-gcomp as a complementary method to estimate the overall associations of serum multivitamin levels with sarcopenia [30]. This method combines the simplicity of WQS with the flexibility of g-computation, allowing individual components in the mixtures to have positive or negative weights. The weights of all compounds sum up to 1. The mixture slope and overall model confidence bounds were iterated by 10,000 bootstraps.

#### Bayesian kernel machine regression

Finally, we utilized the BKMR model, which can estimate the overall relevance of mixed components and evaluate potential interaction and nonlinear associations between multiple exposures [31]. The BKMR model was executed for 10,000 iterations by using the Markov chain Monte Carlo algorithm. The overall association of all vitamins on sarcopenia risk was estimated by calculating the expected change in the outcome for specific percentile exposure levels compared to the median exposure level. The exposure-response curves for each vitamin and their potential interactions with each other were determined by determining the exposure levels of the remaining vitamins at specific percentile levels.

Regarding the sensitivity analysis, we performed stratified and interaction analyses by using baseline features as modifiers to assess potential differences in the association of serum multivitamins across subgroups. All statistical analyses were considered the complex sampling design of NHANES except WQS and BKMR, and were performed with R studio (R Version 4.3.2, Packages “corrplot”, “factoextra”, “rms”, “gWQS”, “qgcomp”, and “bkmr”), *P* < 0.05 was statistically significant.

## Results

### Characteristics of participants

Among the total sample of 5060 adults, 681 (13.46%) individuals were considered to have sarcopenia. The baseline characteristics of individuals with or without sarcopenia are listed in Table 1. Individuals with sarcopenia were more probably to be older, male, Non-Hispanic White, less educated, in poverty status, obese, in drinking state, and had lower physical activity and energy intake (*P* < 0.05). The majority (77.08%) of participants donated blood samples in the daytime. The differences of six-month time period when surveyed were not significant across the sarcopenia stratification groups (*P* = 0.05).

**Table 1:** Characteristics of participants included in NHANES 2003–2006 analyses^a^

Characteristics	Total (*n* = 5060)	Non-sarcopenia (*n* = 4379)	sarcopenia^b^ (*n* = 681)	*P*
Age (years)	44.67 (0.36)	43.60 (0.40)	55.42 (0.79)	< 0.0001
Sex				0.03
Female	2433 (50.26)	2122 (50.88)	311 (44.09)	
Male	2627 (49.74)	2257 (49.12)	370 (55.91)	
Race				< 0.0001
Mexican American	1073 (7.74)	784 (6.79)	289 (17.17)	
Non-Hispanic White	2648 (74.60)	2348 (75.37)	300 (66.87)	
Non-Hispanic Black	960 (9.49)	934 (10.16)	26 (2.82)	
Other	379 (8.18)	313 (7.68)	66 (13.15)	
Education				< 0.0001
Lower than high school	1292 (15.31)	992 (13.84)	300 (29.99)	
High school	1227 (25.11)	1057 (24.64)	170 (29.76)	
Above high school	2541 (59.59)	2330 (61.52)	211 (40.24)	
PIR	3.16 (0.06)	3.22 (0.06)	2.62 (0.08)	< 0.0001
BMI (kg/m^2^)				< 0.0001
<30	3564 (71.57)	3220 (74.56)	344 (41.70)	
≥30	1496 (28.43)	1159 (25.44)	337 (58.30)	
Drink status				< 0.0001
never	620 (10.86)	516 (10.36)	104 (15.80)	
former	998 (15.19)	791 (13.96)	207 (27.43)	
current	3442 (73.95)	3072 (75.67)	370 (56.77)	
Smoke status				< 0.001
never	2530 (48.64)	2191 (48.77)	339 (47.35)	
former	1269 (24.68)	1042 (23.71)	227 (34.43)	
current	1261 (26.68)	1146 (27.53)	115 (18.22)	
Physical activity level				< 0.0001
Sedentary	1133 (16.13)	875 (14.76)	258 (29.85)	
insufficient	2046 (43.12)	1822 (43.81)	224 (36.15)	
Moderate	877 (19.49)	773 (19.71)	104 (17.28)	
High	1004 (21.26)	909 (21.71)	95 (16.72)	
Diabetes				< 0.0001
no	4606 (93.99)	4067 (95.12)	539 (82.64)	
yes	454 (6.01)	312 (4.88)	142 (17.36)	
Hypertension				< 0.0001
no	3525 (72.99)	3149 (74.82)	376 (54.65)	
yes	1535 (27.01)	1230 (25.18)	305 (45.35)	
Total protein (g/dL)	7.16 (0.02)	7.15 (0.02)	7.21 (0.04)	0.04
Energy intake (kcal)	2250.13 (18.30)	2285.93 (20.72)	1892.07 (38.09)	< 0.0001
Six-month time period when surveyed				0.05
November 1 through April 30	2379 (41.78)	1976 (41.16)	403 (47.97)	
May 1 through October 31	2681 (58.22)	2403 (58.84)	278 (52.03)	
Session of blood sample collection				0.02
Morning	2443 (44.06)	2104 (43.92)	339 (45.55)	
Afternoon	1847 (33.02)	1570 (32.58)	277 (37.36)	
Evening	770 (22.92)	705 (23.50)	65 (17.09)	

Abbreviations: BMI, body mass index; PIR, family income to poverty ratio; SMI, appendicular skeletal muscle index

^a^ Continuous data were displayed as weighted means (standard errors), while categorical variables were exhibited as unweighted numbers (weighted percentages)

^b^ Sarcopenia was defined as SMI < 0.789 for male and SMI < 0.512 for female, based on the Foundation for the National Institutes of Health recommendation

### Distribution of serum vitamins

Distributions of serum concentrations of six vitamins are shown in Table S1. The detection rates for all serum vitamins were more than 85%. Furthermore, the mutual correlations between two vitamin measurements were described in Fig. S1. The highest correlation coefficient is 0.45 (VB9 and VE), indicating a generally weak correlation among the vitamins examined.

### Association between each vitamin concentration and sarcopenia

The associations between each vitamin concentration and sarcopenia risk by the survey-weighted logistic regression models are displayed in Table 2. Higher circulating levels of VE (OR (95% CI): 0.635 (0.433, 0.932)), VC (OR (95% CI): 0.828 (0.693, 0.989)) and VD (*P* for trend = 0.021) were negatively associated with the sarcopenia risk, but these associations were not statistically significant after FDR correction. The results showed that there was no linear correlation between the levels of each serum vitamin and sarcopenia. Furthermore, Fig. S3 displays the potential non-linear associations between single serum vitamin levels and the risk of sarcopenia in the RCS model. A nonlinear association between VA and sarcopenia was observed. A U-shaped dose-response relationship was observed between serum vitamin D levels and sarcopenia (*P* for nonlinearity < 0.05). The OR of sarcopenia decreased with increasing VD concentration for participants when serum VD levels of 9.10 to 68.72 nmol/L. Such negative correlation changed when VD concentration was greater than 68.72 nmol/L, indicating the threshold near 68.72 nmol/L of VD concentration.

**Table 2:** Multivariate adjusted odd ratios (95% CI) of Sarcopenia risk with single and multivitamin concentrations among participants in the NHANES 2003–2006*

Vitamins	Continuous	*P*	*P*_FDR_	Q1	Q2	Q3	Q4	*P* for	*P*_FDR_
	OR (95% CI)			(ref)	OR (95% CI)	OR (95% CI)	OR (95% CI)	trend	
VA	0.683 (0.362, 1.286)	0.191	0.286	1	0.774(0.428, 1.402)	0.536(0.287, 1.000)	0.651(0.378, 1.120)	0.113	0.226
VE	0.635 (0.433, 0.932)	**0.027**	0.123	1	0.897(0.485, 1.659)	0.838(0.436, 1.610)	0.671(0.381, 1.183)	0.083	0.226
VB9	1.074 (0.728, 1.585)	0.668	0.802	1	0.722(0.435, 1.199)	0.815(0.396, 1.679)	0.942(0.589, 1.508)	0.978	0.978
VB12	0.984 (0.720, 1.346)	0.906	0.906	1	1.015(0.682, 1.510)	1.038(0.650, 1.659)	1.039(0.655, 1.647)	0.805	0.966
VC	0.828 (0.693, 0.989)	**0.041**	0.123	1	1.071(0.646, 1.775)	0.975(0.562, 1.692)	0.685(0.432, 1.086)	0.168	0.252
VD	0.589 (0.317, 1.098)	0.083	0.166	1	0.641(0.386, 1.065)	0.530(0.318, 0.884)	0.533(0.288, 0.984)	**0.021**	0.126
Multivitamins				1^a^	0.626(0.393, 0.996)^b^	0.580(0.396, 0.848)^c^		**0.002**	–

Abbreviations: Continuous, log-transformed concentration of vitamins; Q, quartile; *P*_*FDR*_, *P* were FDR-adjusted

“–”: means *P*_*FDR*_ was not available

^a^ low-level exposure group; ^b^ middle-level exposure group; ^c^ high-level exposure group

* Models were adjusted for age, sex, race, PIR, education level, smoking status, drinking status, BMI, physical activity level, serum total protein, energy intake, hypertension, diabetes, six-month time period when surveyed and session of blood sample collection

### Association between serum multivitamin concentrations and sarcopenia by K-means

The K-means clustering method clustered all 5060 participants into three clusters, which can be seen in Fig. S2. Cluster 1 was labeled as the “low-level exposure group”, Cluster 2 as the “middle-level exposure group”, and Cluster 3 as the “high-level exposure group”. Table S2 and S3 displays statistics illustrating the distributions of standardized log-vitamin concentrations and the specifics regarding naming clusters. We included the identified cluster patterns in the weighted logistic regression model. Table 2 shows that compared to the low-level group of multiple vitamins, participants with higher levels of vitamins demonstrated a tendency towards lower risks of sarcopenia after adjusting covariates (the 3rd Cluster (OR (95% CI): 0.580 (0.396, 0.848); *P* for trend = 0.002).

### Association between serum multivitamin concentrations and sarcopenia by WQS regression

As demonstrated in Table 3 and Fig. S4, WQS regression analysis showed that serum multivitamin concentrations correlate negatively with sarcopenia (OR (95% CI): 0.768 (0.653, 0.903)). Vitamins D and C were predominant members for the negative association with sarcopenia (weights of 0.673 and 0.161, respectively). No significant findings were observed in the positive WQS regression analysis of serum concentrations of different or multiple vitamins and sarcopenia risk (OR (95% CI): 0.886 (0.746, 1.053)).

**Table 3:** Summary results from different models^*^

Vitamin	Multivariate logistic regression^a^	WQS (positive direction)	WQS (negative direction)	Qgcomp^b^	BKMR
VA		0.151	0.063	0.174 (-)	0.111
VE		0.054	0.161	0.209 (-)	0.109
VB9		0.431	0.007	0.668 (+)	0.083
VB12		0.273	0.022	0.331 (+)	0.030
VC		0.087	0.073	0.275 (-)	0.036
VD		0.004	0.673	0.342 (-)	0.635
Multivitamins	0.580(0.396, 0.848)	0.886 (0.746, 1.053)	0.768 (0.653, 0.903)	0.835 (0.739,0.943)	

Abbreviations: WQS, Weighted Quantile Sum; Q-gcomp, Quantile g-computation; BKMR, Bayesian kernel machine regression; PIP, posterior inclusion probability

* OR (95% CI) was used to assess the association of serum multivitamin levels with sarcopenia

^a^ Compared with participants in the lowest quartile of measured vitamin, participants in the highest quartile of measured vitamin was significantly associated with sarcopenia risk with adjustment for all covariates

^b^ “+” means positive weight while “-” means negative weight

### Association between serum multivitamin concentrations and sarcopenia by Q-gcomp

Consistent with the results in WQS models, the serum concentrations of different or multiple vitamins showed a significantly inverse association with sarcopenia risk in Q-gcomp analyses (OR (95% CI): 0.835 (0.739,0.943), Fig. S5). VD and VC had the greatest negative contribution to the overall inverse associations (34.2%, 27.5% for sarcopenia, respectively).

### Association between serum multivitamin concentrations and sarcopenia by BKMR

The BKMR analyses further identified that the overall association of serum concentrations of different or multiple vitamins was negatively associated with sarcopenia. Compared to the equivalent median, concentrations of serum multivitamin from the 25th to 75th percentile were negatively connected with sarcopenia (Fig. S6a). VD and VA exhibited higher posterior inclusion probability (PIP: 0.635 and 0.111, respectively) as determined by the BKMR models (Table 3). Fig. S6b shows the exposure-response function trend of each serum vitamins. We further explored six serum vitamin interactions, the slopes of the exposure-response function were similar across different quantiles, indicating that there may be no interaction between the different vitamins (Fig. S6c).

### Sensitivity analysis

Subgroup analyses stratified by age, sex, race, drinking and smoking status, diabetes, and hypertension were performed in Table S4. No significant interactions were observed between stratified variables and serum multivitamin concentrations concerning sarcopenia (all *P* for interactions > 0.05). The association between serum multivitamin levels and sarcopenia remains consistent across different categories of individuals.

## Discussion

Sarcopenia is an increasingly recognized musculoskeletal condition associated with significant adverse health outcomes [6]. Our examination of data from 5,060 individuals reveals consistent evidence from four mixed-exposure analytic models, demonstrating an inverse relationship between serum concentrations of different or multiple vitamins and the risk of sarcopenia. Furthermore, we discerned non-linear dose-response curves linking serum levels of vitamins A and D to sarcopenia; notably, vitamin D’s relationship is characterized by a U-shaped dose-response curve. These observations confirm the hypothesis of a preventive association between serum multivitamin levels and the risk of sarcopenia. Additionally, the data suggest that a threshold effect may be present in the correlation involving individual serum vitamin D concentrations and the onset of sarcopenia.

Our investigation identified a U-shaped dose-response curve in the association between serum vitamin D (VD) level and sarcopenia. A significant inverse correlation between serum VD concentration and sarcopenia was apparent at levels below 68.72 nmol/L. This is in line with previous research, such as a randomized controlled trial, which pinpointed a beneficial effect of vitamin D on sarcopenia risk among postmenopausal women at serum VD ranges of 37.44 to 42.43 nmol/L [32]. Additionally, Zhang et al. [33] corroborated a U-shaped relationship between serum VD and sarcopenia risk, with optimal vitamin D concentration identified at 38.5 nmol/L within the NHANES 2011–2014 dataset. Complementing these findings, a comprehensive Mendelian randomization analysis indicated an L-shaped pattern: while sarcopenia risk declined with increasing VD levels up to 49.92 nmol/L, no marked protective benefits were seen beyond this threshold [34]. Notwithstanding methodological variances, these discoveries echo the trends we observed in our study.

The linkage between deficient vitamin D status and amplified risk of sarcopenia is scientifically tenable. Empirical studies have seen muscle deterioration and frailty manifest in animal models deprived of vitamin D or with hindered vitamin D functionality [8], illustrating the possible direct implication of vitamin D deficiency in muscle health.This relationship may be attributed to several biological mechanisms. Vitamin D acts by initiating muscle cell differentiation and proliferation through gene expression catalyzed by nuclear vitamin D receptors, subsequently promoting skeletal muscle fiber enlargement [35]. Additionally, it is implicated in pivotal growth-related signaling pathways and in the regulation of the intracellular calcium messenger system, which in turn impacts muscle growth [36]. Beyond muscle physiology, vitamin D levels are implicated in modulating inflammation and insulin secretion, factors that are also recognized to influence sarcopenia onset [37].

Our analysis did not reveal a marked inverse correlation between serum levels of vitamins B9, B12, C, and E, and sarcopenia when evaluating dose-response relationships. This is divergent from previous research where lower serum concentrations of vitamin B12 were notably associated with muscle wasting in older adults (*P* = 0.015) [38]. Experimental work with animal models has indicated that vitamin C supplementation might ameliorate muscle atrophy by curbing the overproduction of reactive oxygen species [15]. Observational studies in humans have identified a positive relationship between serum vitamin C levels and measurements of skeletal muscle mass in mature populations [39]. Moreover, epidemiological evidence suggests dietary vitamin E could influence muscle health [14]. Yet, these discoveries stand in contrast with our current study’s outcomes, which might be influenced by disparities in sample size, ethnic backgrounds, and methodological differences in covariate handling.

Concerning vitamin A, our study discerned a non-linear correlation with sarcopenia. Research involving animal models suggests vitamin A facilitates the myogenic differentiation of skeletal muscle cells [13]. However, population-based studies on this relationship yield varied results. A recent investigation within a Japanese diabetic senior cohort indicated no significant link between vitamin A intake and sarcopenia, which contradicts our observations [16]. These conflicting outcomes may stem from ethnic variation among subjects, alongside differing assessment techniques for both exposure and outcomes. The association between vitamin A and sarcopenia, thus, remains a subject of ambiguity, warranting further exploration through expansive, prospective research endeavors.

Antioxidants such as vitamins A, C, and E are crucial for strengthening oxidative defense across fluids, cells, and tissues [40]. The B vitamins serve as essential cofactors in metabolic pathways involving homocysteine, and significant deficiencies in B12 and B9 can result in heightened homocysteine levels, engendering oxidative stress and compromising vascular cell function [41]. Vitamin D, functioning as a hormone precursor, is implicated in the modulation of inflammatory responses, with active forms of it shown to suppress pro-inflammatory cytokines such as TNF-α, IL-1β, IL-6, IL-8, and IL-12 while simultaneously augmenting the production of the anti-inflammatory cytokine IL-10 [42]. Given that increased inflammation and oxidative stress are pivotal in the onset of sarcopenia, strategies that target pro-inflammatory cytokines and pathways offer potential preventive benefit for the occurrence of sarcopenia [43].

Serum vitamins may exert their physiological functions while interacting with other vitamins. There is evidence suggesting interactions within vitamin metabolism, including the positive impact of the B9 and B12 interplay on metabolic syndrome outcomes [44] and the synergistic antioxidant effect of vitamins C and E [45]. Additionally, physiological functions carried out by vitamins D and A through the vitamin D receptor (VDR) and retinoid X receptor (RXR) respectively could be affected by antagonistic actions, where high levels of vitamin A metabolites may impede the beneficial actions of vitamin D [46]. Despite these insights, our study did not observe significant interactions when vitamins were exposed simultaneously. Further studies are indispensable to elucidate the potential synergistic or antagonistic mechanisms and to establish a dose-response paradigm that outlines the relationship between serum multivitamin levels and sarcopenia risk.

In the present study, we have conducted a rigorous analysis of the NHANES dataset spanning from 2003 to 2006, encapsulating a substantial and randomized sample reflective of the broader US adult populace. To our knowledge, this endeavor represents a pioneering integrative investigation into the nexus between systemic multivitamin profiles and sarcopenia susceptibility. Employing serum vitamin concentrations as our metric for vitamin exposure has provided a more objective and precise quantification than dietary self-reports, thereby enhancing the reliability of our exposure assessment.

Despite these strengths, our study is subject to certain limitations that merit acknowledgment. While this study accounted for various potential confounding factors, including serum proteins and energy intake, a major limitation of this study is the lack of adjustment for other potential dietary risk factors for sarcopenia, such as the roles of vitamin K, trace minerals, and pharmacological interventions. The reliance on a single baseline measurement of serum vitamin concentrations may not accurately reflect long-term nutritional status, which could introduce some degree of misclassification. Additionally, the demographic focus on US adults curtails the global applicability of our findings, as they may not be extrapolated to diverse ethnic groups seamlessly. Furthermore, the cross-sectional design of our analysis imposes inherent constraints on our ability to infer causality between multivitamin co-exposure and the development of sarcopenia.

Given these limitations, future research endeavors should harness longitudinal designs and randomized control trials to affirm these preliminary associations. Such studies would be instrumental in establishing a cause-and-effect paradigm as well as refining our understanding of the dose-response interplay between multivitamin co-exposure and lowering sarcopenia risk.

## Conclusion

The current study investigates the association between serum concentrations of various vitamins, both individually and collectively, and the risk of sarcopenia among adults in the United States. Our results imply that co-exposure to multiple vitamins, with vitamin D being especially significant, may mitigate the risk of developing sarcopenia. However, given the inherent constraints of cross-sectional analysis, it is imperative that future research employ prospective cohort studies and controlled experiments to substantiate these preliminary findings and uncover the mechanisms at play.

## Electronic supplementary material

Below is the link to the electronic supplementary material.

Supplementary Material 1

## Data Availability

The datasets generated and analyzed in the current study are available at NHANES website: https://www.cdc.gov/nchs/nhanes/index.htm.
