# Supplementary material for: Serum concentrations of different or multiple vitamins and Sarcopenia risk among US adults: insights from NHANES
Source: BMC Public Health. 2024 Dec 4;24:3372. doi: 10.1186/s12889-024-20897-9 (PMC11616181; doi:10.1186/s12889-024-20897-9)
Supplement: Supplementary file 1 — Supplementary Material 1 [file 12889_2024_20897_MOESM1_ESM.docx]

**Supplementary materials**

**Table S1** Distributions of vitamins in the study population.

Abbreviations: VA, vitamin A; VE, vitamin E; VB9, vitamin B9; VB12, vitamin B12; VC, vitamin C; VD, vitamin D.

**Table S2** Centres of the four clusters of the six vitamins with prior log-transformation and standardized.

**Table S3** Distributions of standardized circulating levels of the log-transformed vitamins.

The K-means cluster method is a very powerful unsupervised machine learning algorithm, grouping objects into K number of clusters based on minimizing the sum of squares of Euclidean distance between data points and the corresponding cluster centroid.

The K-means clustering method clustered all 5060 participants into three groups based on data points from the six studied vitamins. Concerning the three clusters, we designated the ‘low-level exposure group’ to cluster 1, considering the values of serum vitamin concentrations close to their 25th percentiles, ‘middle-level exposure group’ to cluster 2 for the reason that the values of serum vitamin concentrations close to their 50th percentiles, and ‘high-level exposure group’ to cluster 3 for the reason that the values of serum vitamin concentrations close or over their 75th percentiles.

**Table S4** Subgroups Analysis for the associations of serum multivitamin concentrations with the risk of sarcopenia^*^.

Abbreviations: ^a^ low-level exposure group; ^b^ middle-level exposure group; ^c^ high-level exposure group.

*Models were adjusted for age, sex, race, PIR, education level, smoking status, drinking status, BMI, physical activity level, serum total protein, energy intake, hypertension, diabetes, six-month time period when surveyed, session of blood sample collection.

**Fig. S1** Heat-map illustration of pairwise correlations of circulating levels of six vitamins in serum.

Abbreviations: VA, vitamin A; VE, vitamin E; VB9, vitamin B9; VB12, vitamin B12; VC, vitamin C; VD, vitamin D.

**Fig. S2** Visualisation of K-means clustering of 5060 NHANES participants based on six vitamins in serum. Blue dots refer to cluster 1 (low-level exposure group); yellow dots refer to cluster 2 (middle-level exposure group); gray dots refer to cluster 3 (high-level exposure group).

**Fig.S3** Adjusted exposure-response relationship between log-transformed vitamins and sarcopenia.

**Fig.S4** The WSQ regression estimated weights of each of the six vitamins associated with sarcopenia risk in the positive direction (a) and in the negative direction (b). Models were adjusted for age, sex, race, PIR, education level, smoking status, drinking status, BMI, physical activity level, serum total protein, energy intake, hypertension, diabetes, six-month time period when surveyed and session of blood sample collection.

**Fig. S5** The directions and magnitude of the assigned weights for each log-transformed vitamin concentrations in relation to sarcopenia in quantile g-computation. Models were adjusted for age, sex, race, PIR, education level, smoking status, drinking status, BMI, physical activity level, serum total protein, energy intake, hypertension, diabetes, six-month time period when surveyed and session of blood sample collection.

**Fig. S6** Associations of the serum vitamins with sarcopenia risk estimated by Bayesian Kernel Machine Regression (BKMR). (a) Combined effects of serum vitamins mixture on sarcopenia risk. This plot showed the estimated difference in sarcopenia risk and 95% confidence interval when all vitamin concentrations were held at particular percentiles compared to their medians. (b) Single vitamin-exposure effect (95% CI) to sarcopenia when other vitamins were fixed at a specific quantile (25th, 50th, 75th). (c) Bivariate exposure–response relationship for each vitamin presented on the upper coordinate axis and sarcopenia risk when the corresponding vitamins on the right longitudinal axis were fixed at the 25th, 50th, and 75th percentiles, and the remaining vitamins held at the 50th percentiles.

Models were adjusted for age, sex, race, PIR, education level, smoking status, drinking status, BMI, physical activity level, serum total protein, energy intake, hypertension, diabetes, six-month time period when surveyed, session of blood sample collection.

**Table S1** Distributions of vitamins in the study population.

| Vitamins | LOD | Detection rate (%) | Median | Interquartile range |
| --- | --- | --- | --- | --- |
| VA (μmol/L) | 1.03 μg/dL | 90.38 | 2.01 | 1.67-2.40 |
| VE (μmol/L) | 0.94 μmol/L | 90.38 | 26.94 | 21.60-35.29 |
| VB9 (nmol/L) | 0.11 nmol/L | 88.17 | 25.80 | 18.80-35.95 |
| VB12 (pmol/L) | 14.76 pmol/L | 87.67 | 347.60 | 263.47-458.30 |
| VC (μmol/L) | 1.70 μmol/L | 90.67 | 54.50 | 34.10-69.30 |
| VD (nmol/L) | 3.75 nmol/L | 89.17 | 56.80 | 42.20-72.90 |

Abbreviations: VA, vitamin A; VE, vitamin E; VB9, vitamin B9; VB12, vitamin B12; VC, vitamin C; VD, vitamin D.

**Table S2** Centres of the four clusters of the six vitamins with prior log-transformation and standardized.

| Variables | Cluster 1  (Low-level exposure) | Cluster 2  (Middle-level exposure) | Cluster 3  (High-level exposure) |
| --- | --- | --- | --- |
| VA | -0.694 | 0.096 | 0.513 |
| VE | -0.593 | -0.270 | 0.984 |
| VB9 | -0.765 | -0.148 | 0.959 |
| VB12 | -0.280 | -0.262 | 0.673 |
| VC | -0.776 | 0.093 | 0.594 |
| VD | -1.033 | 0.363 | 0.422 |

**Table S3** Distributions of standardized circulating levels of the log-transformed vitamins.

| Variables | Min | 25^th^ percentile | Median | 75^th^ percentile | Max |
| --- | --- | --- | --- | --- | --- |
| VA | -19.009 | -0.585 | 0.043 | 0.634 | 3.597 |
| VE | -12.577 | -0.651 | -0.112 | 0.547 | 4.579 |
| VB9 | -5.474 | -0.648 | -0.028 | 0.616 | 8.008 |
| VB12 | -5.501 | -0.606 | -0.030 | 0.546 | 10.386 |
| VC | -5.895 | -0.355 | 0.288 | 0.617 | 2.503 |
| VD | -4.366 | -0.618 | -0.031 | 0.718 | 3.159 |

The K-means cluster method is a very powerful unsupervised machine learning algorithm, grouping objects into K number of clusters based on minimizing the sum of squares of Euclidean distance between data points and the corresponding cluster centroid.

The K-means clustering method clustered all 5060 participants into three groups based on data points from the six studied vitamins. Concerning the three clusters, we designated the ‘low-level exposure group’ to cluster 1, considering the values of serum vitamin concentrations close to their 25th percentiles, ‘middle-level exposure group’ to cluster 2 for the reason that the values of serum vitamin concentrations close to their 50th percentiles, and ‘high-level exposure group’ to cluster 3 for the reason that the values of serum vitamin concentrations close or over their 75th percentiles.

**Table S4** Subgroups Analysis for the associations of serum multivitamin concentrations with

the risk of sarcopenia*.

| Character | Cluster 1^a^ | Cluster 2^b^ | Cluster 3^c^ | *P* for trend | *P* for interaction |
| --- | --- | --- | --- | --- | --- |
| Age (years) |  |  |  |  | 0.563 |
| 20-39 | ref | 0.646 (0.256, 1.633) | 0.306 (0.060, 1.573) | 0.123 |  |
| 40-59 | ref | 0.601 (0.296, 1.217) | 0.560 (0.303, 1.034) | 0.072 |  |
| ≥60 | ref | 0.750 (0.335, 1.682) | 0.776 (0.371, 1.625) | 0.549 |  |
| Sex |  |  |  |  | 0.495 |
| Female | ref | 0.708(0.394,1.274) | 0.477(0.232, 0.978) | 0.039 |  |
| Male | ref | 0.567(0.327, 0.982) | 0.670(0.416, 1.079) | 0.119 |  |
| Race |  |  |  |  | 0.614 |
| Mexican American | ref | 0.844 (0.542, 1.313) | 0.972 (0.493, 1.914) | 0.847 |  |
| Non-Hispanic White | ref | 0.641 (0.335, 1.226) | 0.570 (0.343, 0.945) | 0.031 |  |
| Non-Hispanic Black | ref | 0.485 (0.027, 8.764) | 0.214 (0.026, 1.769) | 0.086 |  |
| Other | ref | 0.470 (0.156, 1.421) | 0.363 (0.073,1.819) | 0.139 |  |
| Smoke status |  |  |  |  | 0.067 |
| never | ref | 0.424 (0.235, 0.765) | 0.477 (0.270, 0.842) | 0.028 |  |
| former | ref | 0.748 (0.312, 1.795) | 0.514 (0.210, 1.255) | 0.079 |  |
| current | ref | 0.711 (0.327, 1.549) | 1.249 (0.488, 3.198) | 0.861 |  |
| Drink status |  |  |  |  | 0.674 |
| never | ref | 0.227 (0.059,0.866) | 0.238 (0.051, 1.102) | 0.077 |  |
| former | ref | 0.768 (0.276, 2.141) | 0.455 (0.232, 0.893) | 0.017 |  |
| current | ref | 0.663 (0.384, 1.146) | 0.685 (0.427, 1.097) | 0.12 |  |
| Hypertension |  |  |  |  | 0.444 |
| no | ref | 0.597 (0.342, 1.042) | 0.514 (0.281, 0.941) | 0.034 |  |
| yes | ref | 0.708 (0.361, 1.390) | 0.661 (0.339, 1.286) | 0.21 |  |
| DM |  |  |  |  | 0.195 |
| no | ref | 0.665 (0.421, 1.052) | 0.633(0.425, 0.943) | 0.035 |  |
| yes | ref | 0.433 (0.167, 1.124) | 0.312 (0.102, 0.959) | 0.046 |  |

Abbreviations: ^a^ low-level exposure group; ^b^ middle-level exposure group; ^c^ high-level exposure group.

*Models were adjusted for age, sex, race, PIR, education level, smoking status, drinking status, BMI, physical activity level, serum total protein, energy intake, hypertension, diabetes, six-month time period when surveyed, session of blood sample collection.


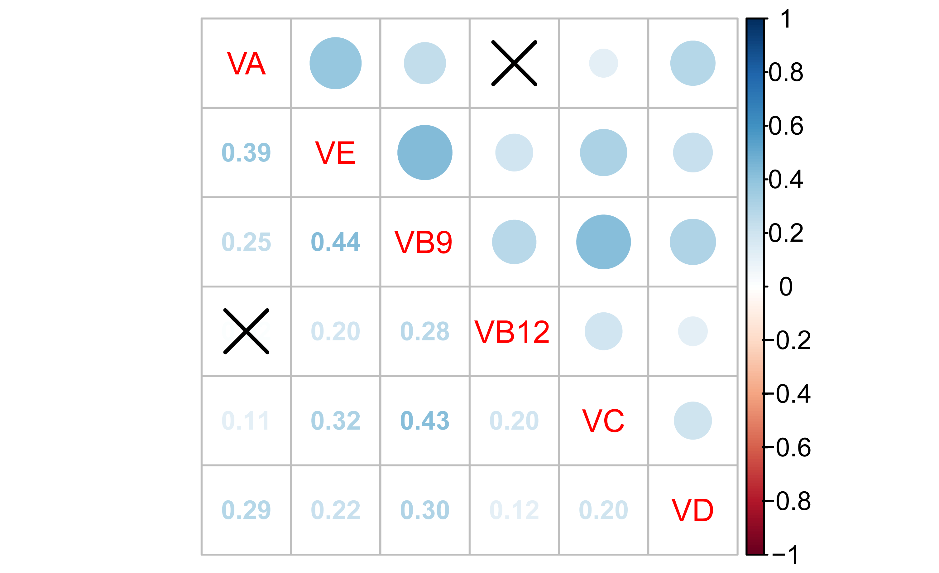


**Fig. S1** Heat-map illustration of pairwise correlations of circulating levels of six vitamins in serum.

Abbreviations: VA, vitamin A; VE, vitamin E; VB9, vitamin B9; VB12, vitamin B12; VC, vitamin C; VD, vitamin D.

**
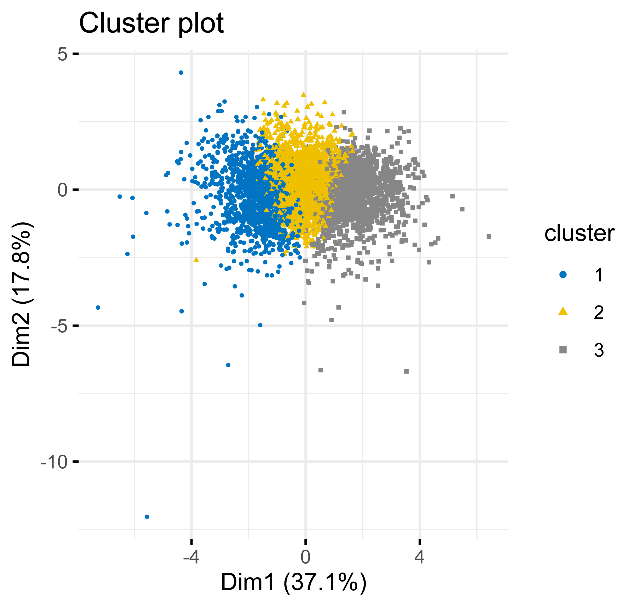
**

**Fig. S2** Visualisation of K-means clustering of 5060 NHANES participants based on six vitamins in serum. Blue dots refer to cluster 1 (low-level exposure group); yellow dots refer to cluster 2 (middle-level exposure group); gray dots refer to cluster 3 (high-level exposure group).


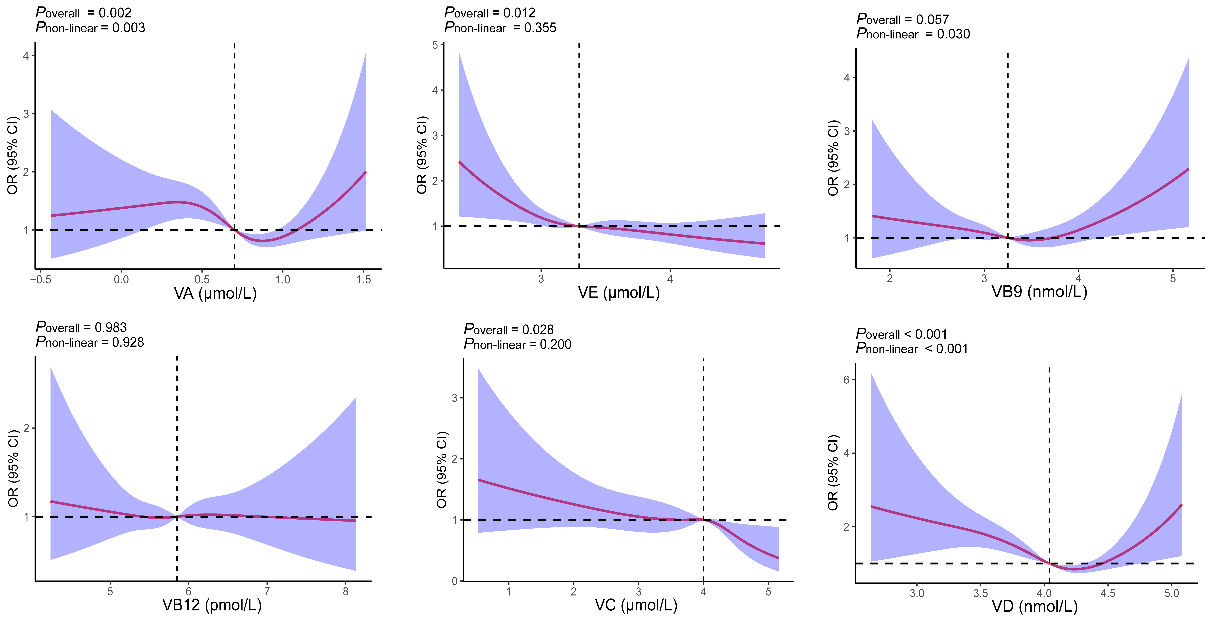


**Fig.S3** Adjusted exposure-response relationship between log-transformed vitamins and sarcopenia.


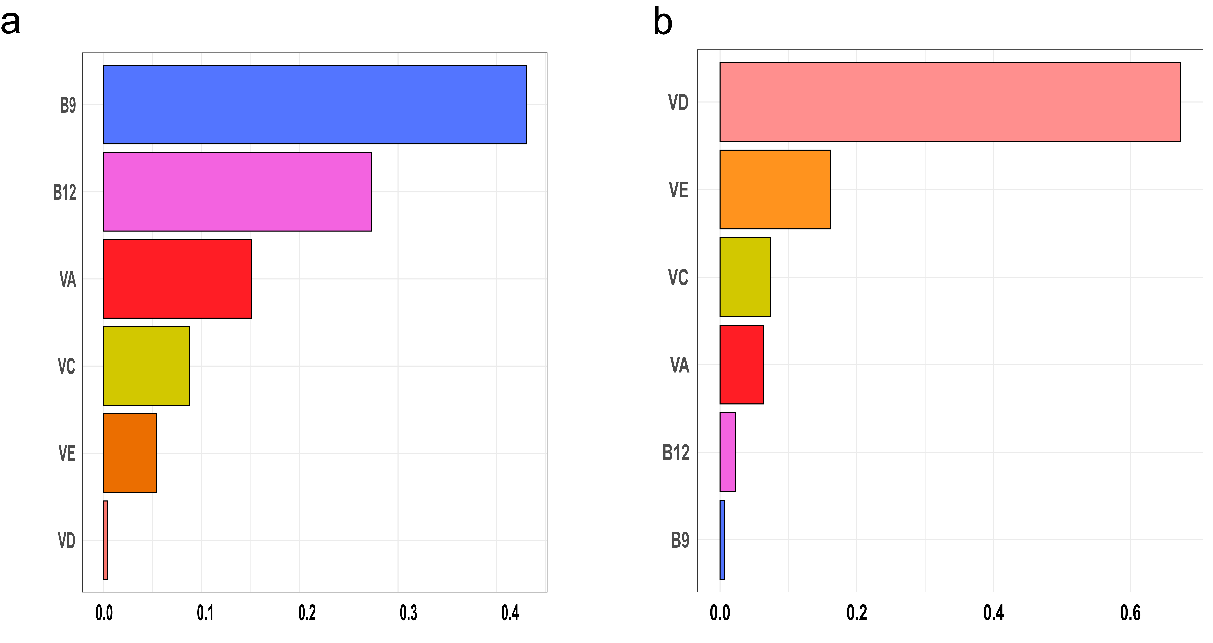


**Fig.S4** The WSQ regression estimated weights of each of the six vitamins associated with sarcopenia risk in the positive direction (a) and in the negative direction (b). Models were adjusted for age, sex, race, PIR, education level, smoking status, drinking status, BMI, physical activity level, serum total protein, energy intake, hypertension, diabetes, six-month time period when surveyed and session of blood sample collection.


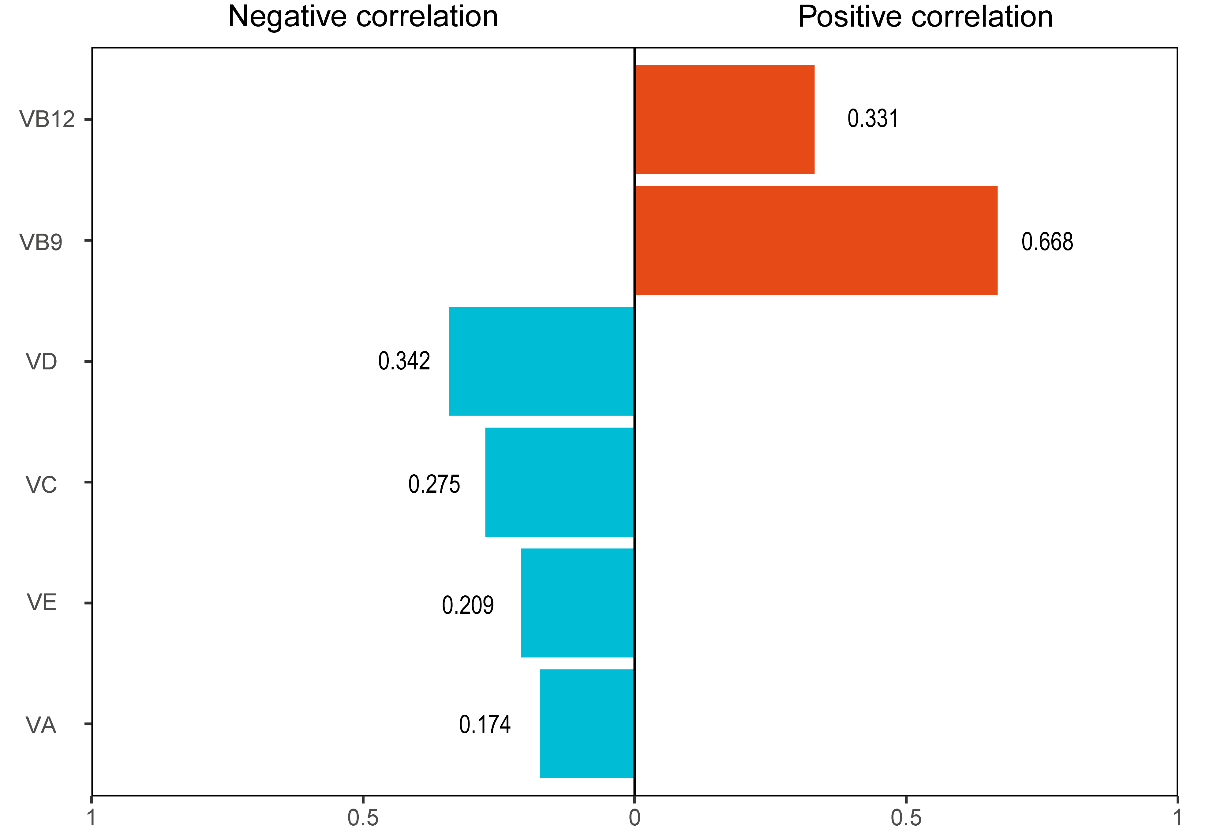


**Fig. S5** The directions and magnitude of the assigned weights for each log-transformed vitamin concentrations in relation to sarcopenia in quantile g-computation. Models were adjusted for age, sex, race, PIR, education level, smoking status, drinking status, BMI, physical activity level, serum total protein, energy intake, hypertension, diabetes, six-month time period when surveyed and session of blood sample collection.


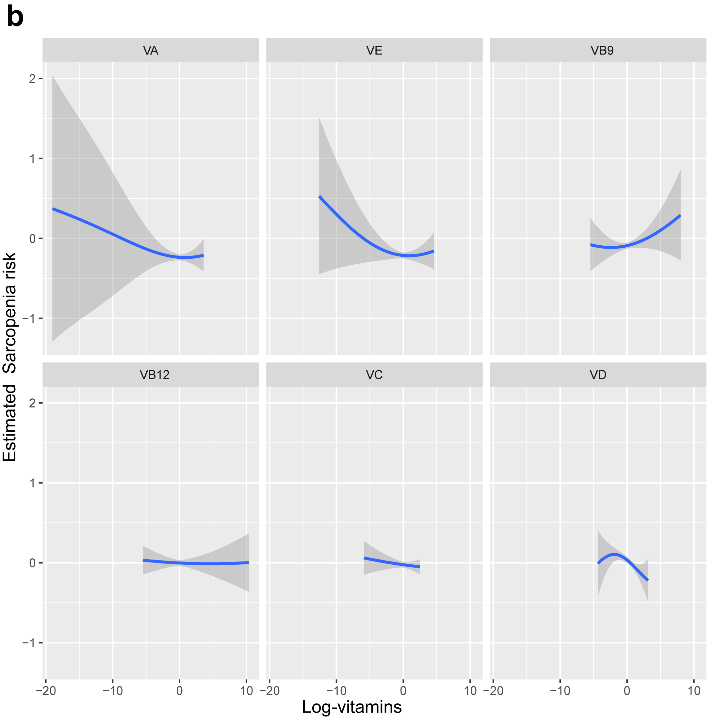

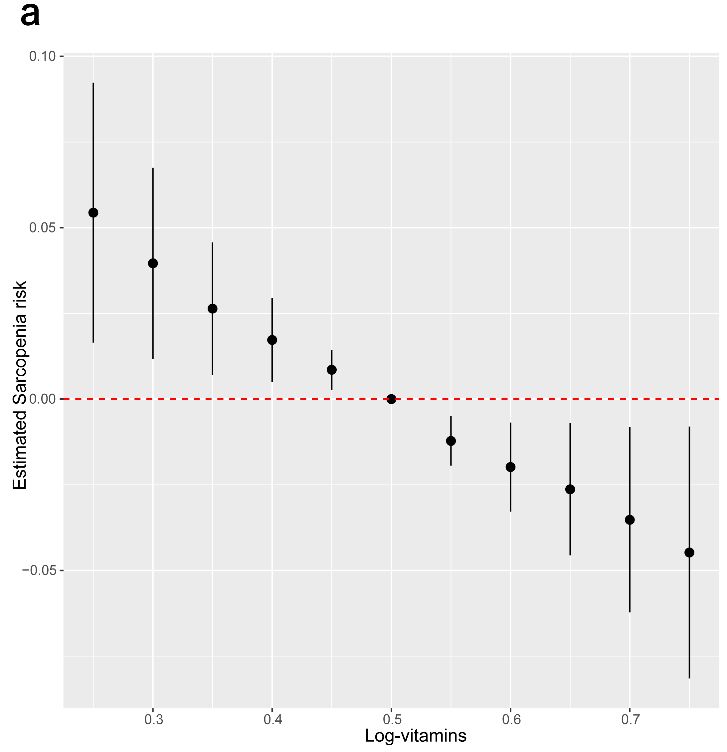


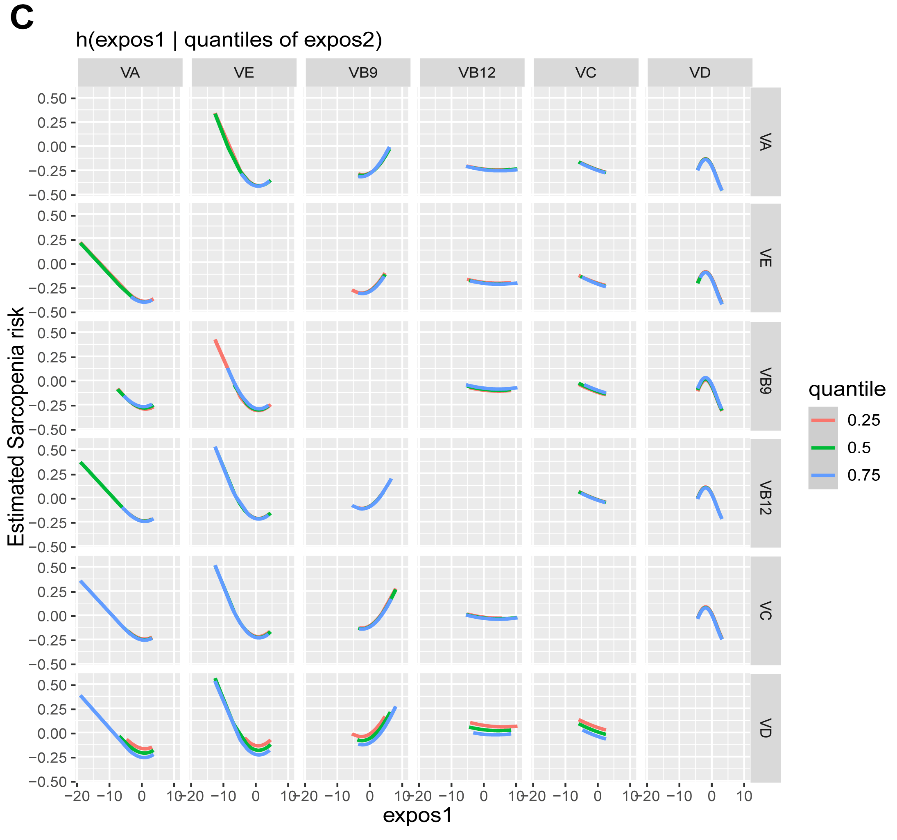


**Fig. S6** Associations of the serum vitamins with sarcopenia risk estimated by Bayesian Kernel Machine Regression (BKMR). (a) Combined effects of serum vitamins mixture on sarcopenia risk. This plot showed the estimated difference in sarcopenia risk and 95% confidence interval when all vitamin concentrations were held at particular percentiles compared to their medians. (b) Single vitamin-exposure effect (95% CI) to sarcopenia when other vitamins were fixed at a specific quantile (25th, 50th, 75th). (c) Bivariate exposure–response relationship for each vitamin presented on the upper coordinate axis and sarcopenia risk when the corresponding vitamins on the right longitudinal axis were fixed at the 25th, 50th, and 75th percentiles, and the remaining vitamins held at the 50th percentiles.

Models were adjusted for age, sex, race, PIR, education level, smoking status, drinking status, BMI, physical activity level, serum total protein, energy intake, hypertension, diabetes, six-month time period when surveyed, session of blood sample collection.
